# Association between left atrial sphericity index and clinical outcomes in patients with systolic heart failure

**DOI:** 10.1002/clc.23562

**Published:** 2021-02-04

**Authors:** Mimi Chapman, Teruhiko Imamura

**Affiliations:** ^1^ Second Department of Internal Medicine University of Toyama Toyama Japan

To the editor

Left atrial remodeling is receiving great concern as a great contributor to the heart failure progression in addition to the biventricular function.^1^ However, the optimal methodology to assess the degree of left atrial remodeling has not yet been established thus far. Yazaki et al. demonstrated that the left atrial sphericity index, instead of left atrial emptying fraction and left atrial volume, was an independent predictor of heart failure hospitalization among those with systolic dysfunction.^2^ Several concerns should improve their findings.

The primary concern is the variability of this index. In general, left atrial size is variable depending on hemodynamic status. Data on which timings (on admission, at discharge, or stable ambulatory setting) were the index measured would strengthen their findings. If the index is variable, the next concern is the possibility of intervention on this index to improve clinical outcomes. For example, heart rate modulation, preload unloading by using diuretics, and afterload reduction by using vasodilators might improve the index and clinical outcomes.

The second concern is the mechanism of worsening heart failure in patients with higher sphericity index: whether the index is a marker of poor background and/or a contributor to the worsening heart failure. Detailed hemodynamic assessment using invasive right heart catheterization, histopathological assessment of endomyocardium, and measurement of peripheral inflammatory markers would further approach the mechanism.

As minor concerns, their hypothesis might be expanded into those with cardiac diastolic dysfunction.^1^ Optimal cutoff of sphericity index, which is calculated using receiver operating characteristics analysis, might be more accurate instead of just using median value.

## Data Availability

Data sharing is not applicable to this article as no new data were created or analyzed in this study.
